# Eighteenth Annual Meeting of the American Medical Association

**Published:** 1867-06

**Authors:** 


### Miscellaneous.

### Eighteenth Annual Meeting of the American Medical Association.

The Eighteenth Annual Meeting of the American Medical Association commenced on the 7th of May, at Hopkins's Music Hall, Cincinnati, and was called to order at 11 o'clock, by the President, Dr. Henry F. Askew.

After prayer by Rev. Dr. Storrs, Dr. John A. Murphy, Chairman of the Committee of Arrangements, bade the Association welcome in a brief and appropriate speech.

*President's Address.*—Dr. Askew delivered his annual address. Its tone was deprecatory, rather than laudatory, of the progress of the Association, as manifested in the circulation of the annual reports, which contained valuable knowledge and able papers on subjects interesting to the profession. The membership was three thousand; but the attendance only a tenth of that number. From a thousand to eleven hundred copies of the annual report formerly printed, the number has fallen to six hundred, and these include the copies sent gratis to medical and other journals. Renewed efforts should be made to increase the demand for the reports.

The address next discussed the restrictions of membership, and invited attention to the subject. Specialists, and their efforts to obtain notoriety by advertising, were noticed. The diploma may once have been necessary to vouch for the doctor, but now it is only regarded as a certificate that he has completed his studies, and is qualified to take charge of the life of his patients. The danger in special practice is, that in the disturbance of a part the whole must be affected, and hence the necessity that the patient should have the attention of the general, as well as the special practitioner.

The influence of the Association is to check empiricism and to disseminate the best knowledge among the profession, but not until the schools consent that pupils study and master the elementary branches can the profession attain to perfection. The thoroughness of the European schools was commended.

The subject of opium-eating was noticed next. The speaker stated that its use was almost as extensive and its effect as alarming as that of alcohol. Calls on the apothecary for it were constant. Children were sent for it to the drug store and received it in quantities incredible to those not acquainted with the traffic. The honor, interest and respectability of the profession would be benefited by action to prevent the abuse of opium.

The address touched on other subjects interesting to the Association, and a copy was requested for publication.

*Report upon the Naval Medical Staff Rank.*—This report was read by Dr. Pinkney, who holds the rank of Commodore in the navy, and appreciates most fully the wrongs of the medical profession in the United States Navy. The following passage will serve to illustrate its character:

"Our service is overgrown with usages which sprung up in the earlier and ruder ages of naval life, and still cling to it with a power and tenacity which almost defy modern enlightenment, progress, and even law. It is probable that the National authorities, who organized the existing rank of medical officers, intended to confer a more substantial fact than the usages of ship-board life have permitted. Among the usages of the service, is that which limits an officer's rights and comforts to the apartments in which he messes, even though his rank actually entitles him to higher privileges and greater comforts than belong to those of an inferior rank, who make up the majority of the inmates of that apartment.

The steerage is the most humble of those apartments, and is the dwelling-place of the very young, or those of no responsibility. The ward-room gathers in it most of the commissioned, and some warrant officers, and was originally occupied by none of higher rank than Lieutenant. All its usages and government are still conformed to the scale of that grade.

"Now, make a medical officer in name an Admiral, and leave him to be ward-room officer, and the title becomes ridiculous. It is sunk below the usages and restrictions originally designed for those of junior years and of inferior rank.

"There is only one mess which is superior to these restrictions, and that is the mess or messes of the commanding officers and their associates, who may range in rank from a Lieutenant Commander to an Admiral. Sometimes there are one, sometimes two of these messes. This is very properly left to the will of the Commander-in-chief, who may choose that he and his Captains may have one or the separate establishments. The Assistant-Surgeon enters the service with the rank of Master. That this title may not be misunderstood, it may be necessary to explain that it is the lowest rank in the ward-room, for the incumbent is, in modern times, generally a graduate of the Naval Academy, awaiting his promotion to Lieutenancy. Like the Master, the Assistant-Surgeon at once becomes a member of the ward-room mess, and unless the number of partitioned-off sleeping-berths contained in the ward-room are occupied by his seniors, he may have the good fortune to occupy one of those that are dimly lighted by an air-port, six inches in diameter. This space is so restricted, and the separation from the common apartment is so slight, that words in an ordinary voice in another become common property."

After further presenting the discriminations against medical men, in regard to ship-board accommodations, Commodore Pinkney said:

"The general law is that no officer shares in prize money unless his name be borne upon the books of the vessel making the captures; but the Admiral or Commander-in-chief has a percentage on all prizes made. The Fleet-Surgeon, as a member of the Commander-in-chief's staff must be with him in the flag-ship, and, as a rule, at the post of the greatest risk, responsibility and hazard, consequently he is not likely to have his name borne upon the books of the subordinate vessels making captures, and yet no share of prize money is allowed him."

The report suggests the following as the remedy for these evils:

"1.—After they have reached the rank of Commander, or are filling the position of Fleet-Surgeon, let them be by right, as they often have been by courtesy, members of the cabin mess. If the mess of the Commander in-chief be too exalted a social position for the members of your profession who are filling the important position of Fleet-Surgeon, then let them be members by right of the mess of the Commander of the ship and the Fleet-Captain.

"2.—An equitable arrangement of prize money, most important in principle, your Committee hope to see effected. It will, however, require future legislation."

In European countries, the Doctor said, more liberal regulations prevail with regard to naval surgeons than in democratic America.

"The late Admiral Foote, so justly distinguished for his large-minded liberality, advocated the highest rank for naval medical officers. An Admiral, among the most distinguished in the service, has authorized to be officially said that he thought the Fleet-Surgeon should in our service, as in the French, be a member of the Commander-in-chief's staff and family. We regard it as opposed to the public interests of the service, which can never be sacrificed to gross indignity without detriment."

Commodore Pinkney offered in conclusion a resolution that a committee of five be appointed by the Chair to present the subject to the President of the United States and to the Secretary of the Navy. Carried unanimously.

On motion, it was resolved to memorialize Congress to enact a law giving a proper share of prize money to medical naval officers.

The Secretary announced several papers, which were referred to sections, and will come up in regular order hereafter.

The report of the Committee on Publication was read.

The Association refused to abolish the payment of money for prize essays, and substitute a certificate and a hundred printed copies of the essays to the essayist.

On motion it was resolved that the delegates from the different States should meet on the 8th, and agree among themselves upon their candidates on the new committee, and report the same to the Secretary.

The Association then adjourned to meet at the same place at 9 o'clock Wednesday morning.

---

SECOND DAY.

The American Medical Association met at Hopkins's Hall at 9 o'clock, and resumed its business.

Minutes of the first session were read and approved.

*Report of Delegates to the Foreign Medical Association.*—The report of the Committee on the above was read by Dr. C. C. Cox. Report referred to the Committee on Publication.

The Chair appointed the following delegates to the Foreign Medical Association for the present year:—B. Fordyce Barker, New York; John E. Tyler, Massachusetts; Thomas C. Brinsmade, Troy, N. Y.

*Reports of Committees.*—The report of Dr. Walker, who presented Dr. Ray's report on Insanity, was made the special order for Thursday, at 10 o'clock A. M.

The Chair announced the Committee on Medical Rank in the United States Navy as follows:—N. S. Davis, Illinois; J. M. Toner, District of Columbia; S. D. Gross, Pennsylvania; J. J. Cockerill, Maryland. H. F. Askew, Delaware.

Dr. Gross read his report on Medical Education. The report is lengthy, and is an embodiment of the action of the Medical College Convention, already published in our last *Journal.*

Dr. Davis read a summary of the proceedings of the Convention of Medical Teachers, for the information of the Association. He stated that the discussions were most thoroughly conducted, and were characterized by the best of feelings. He read at length the report of the Business Committee.

The report was, on motion, referred to the Committee on Publication.

The Committee on Prize Essays reported the reception of eight essays, and the selection of two. That selected for the First Prize was "On the Cause of Intermittent and Remittent Fevers"—the motto of which was, "*Fortis est veritas.*" The Second Prize was assigned to an essay "On the Treatment of certain Abnormities of the Uterus," the motto being "Empiricism in medicine and surgery is fast giving way to the rationalism of true diagnosis."

The envelopes enclosing the names of the successful competitors were then opened, and the following announcements made:—First Prize—To J. R. Black, M. D., of Newark, Ohio. Second Prize—To Montrose A. Pallen, M. D., of St. Louis, Mo.

Report referred to Committee on Publication.

Dr. Sayre, of New York, offered the following:

"*Resolved*, That this Association most cordially approve of the whole action of the Convention of Delegates from the Medical Colleges, assembled in Cincinnati, May 3d, 1867, and urge its practical adoption by all the medical colleges in our country."

After a brief debate, in which Drs. Post, Davis and Lee participated, the motion was carried.

It may be stated here that the report of the Treasurer, read yesterday, showed the Association to be in debt $196, and that from year to year this condition is the same.

Dr. Stillé presented the report of the action of the Convention of Medical Teachers held at the Medical College, which was received.

Dr. Atkinson called up the resolution abolishing the payment of prizes in future.

Dr. Davis objected to the passage of the resolution, which virtually repudiated the payment of future prizes. As the treasury would soon again be full, he would call the attention of the Association to the fourth section of the by-laws. He considered that the transactions called for more original papers at the expense of bulk. The matter should be better, even if the volume be smaller.

Dr. Bibbins, of New York, called attention to a parliamentary point. He favored a more careful consideration of the subject.

Dr. Bronson, of Massachusetts, was opposed to prospective action.

Dr. Atkinson called attention to the fact that the Committee were last year obliged to hold themselves personally responsible for the debt. He said that the present funds of the treasury were even now insufficient to print the matter already in their possession.

Dr. Bronson thought that the fault lay with the different sections, who did not exercise the requisite discrimination.

Dr. Bibbins called the attention of the Chair to the fact that the resolution was in conflict with the by-laws.

Dr. Toner considered that assessments would meet all the necessities of the case. He offered the following:

"*Resolved*, That all members yearly pay five dollars, and that the names of those failing to pay, at the end of three years, be designated in the catalogue by a star or cross."

Dr. Sayre moved as an amendment, that the proposed action of the Association be published in the various medical journals.

After a spirited debate, in which several delegates joined, a motion of Dr. Davis to lay the original motion on the table prevailed.

Dr. Robbins offered the following:

"*Resolved*, That hereafter the Committee of Arrangements be directed to have the ordinances governing the Sections printed on slips and distributed at the several places where the Sections meet." Carried.

The following papers were read and disposed of:—Observations on Diseases of the Throat, as seen in the Military Service, from 1861 to 1865. By Prof. M. K. Taylor, M. D. Referred to Section on Practical Medicine.

A Novel Case of Lithotomy. By D. E. Whinney, of Iowa. Referred to Section on Surgery.

Ligature of the Subclavian Artery. By Willard Parker, M. D., of New York. Referred to Section on Surgery.

The Secretary read a communication offering amendments to the Constitution, which was laid over for one year, as provided for by the Constitution.

A paper was read by Dr. B. Howard, of New York—before the Surgical Section of the Academy—entitled "Ligation, with depletion, of Varicose Veins of the Leg, with a case."

Dr. Cox presented advance proof-sheets of "Provisional Nomenclature of Disease," which was published in London. Referred to Section on Practical Medicine. Also another "On Compulsory Vaccination," by Dr. A. N. Bell, of Brooklyn, N. Y., was presented by deputy, and, on motion, referred to Committee on Hygiene.

Dr. Hammar, of Missouri, offered certain resolutions bearing upon certain irregularities in the profession, which was referred to Committee on Medical Education.

Dr. Gilbert, of New York, exhibited an instrument for the protection of the periosteum in excisions, etc. Referred to Section on Surgery.

Dr. Post read the report of the Committee on Medical Literature, in which he gave certain bibliographical items.

*Medical Statistics in the Army.*—Dr. Benjamin Howard, of New York, offered the following preamble and resolutions:

"*Whereas*, There has been issued, and still remains in force, an official order from the Surgeon-General of the United States Army, prohibiting the communication of any medical or surgical information by any medical officer of the United States Army, to any person whatsoever, without special permission from the Medical Bureau at Washington—thus appropriating, as far as the official power of the Surgeon-General can compass it, all the valuable experience and statistics of all medical men who have served in the various departments of the United States Army, to the exclusive use of the Medical Bureau. And,

"*Whereas*, Under such arbitrary control, an official report has already been made tending to create incorrect impressions on scientific questions of great practical importance to the profession and to society. And,

"*Whereas*, It is important to the reputation of all medical men who served during the war, that they have the opportunity of correcting such erroneous impressions by an examination of the original records. Therefore, be it

"*Resolved*, That it is the opinion of this Association that the monopoly now exercised by the Medical Bureau over the medical and surgical records of the war, is contrary to the genius and catholic spirit of our profession, and obstructive to the highest interests of science and humanity.

"*Resolved*, That the Secretary of War, or other proper authorities, be requested to direct that the original records of the Medical and Surgical History of the War be rendered accessible on certain regular days of each month for purposes of scientific investigation to all medical men who have served as such in the Army of the United States."

Dr. Howard spoke at length in support of the resolution. He dwelt upon the jealousy of departments against interference on the part of outsiders. He did not aim at any one in particular as a target; he spoke for the benefit of his profession.

Dr. Woodward asked for the reading of the preamble and resolution. He then objected to the ventilation of private grievances on the part of any member. The Surgeon-General of the Army, a most efficient officer, was striving to do his duty to the profession; he did not wish those who had contributed nothing to interrupt the business of his bureau for searches. Besides that, the records of the office were now being consulted for the adjustment of pension claims—indiscriminate disturbance of these records was out of the question. He therefore moved that the resolution be laid upon the table, which was carried by a very decided vote.

After the reading of the Report on Medical Literature, Dr. Sayre protested against certain portions as being too highly laudatory of the Board of Health of New York, and as containing matter foreign to the scope of such a report. He also made some remarks upon cholera and quarantine, and claimed that there were few if any cases of that disease to combat, and that these same had been introduced by breakages of the quarantine.

Dr. Davis supported the spirit of the Committee's report, and moved that it take the usual course of reference to the Committee on Publication, which was adopted.

Dr. Hibberd offered the following:

"*Resolved*, That the habit of using unofficinal preparations of medicine by physicians, except where there is no officinal preparation that will answer the purpose as well, is unscientific and imprudent, tending to demoralize the therapeutist and encourage irregular pharmaceutists and nostrum makers, and should be abandoned.

"*Resolved*, That the profession should not patronize druggists who are engaged in the manufacture of nostrums."

On motion, tabled. The Association then adjourned.

---

### Third Day.

The Association met again on the 9th, the hall being full, though not as crowded as on the 8th. President Askew called the meeting to order.

Dr. D. H. Storer arose to a question of privilege, namely, the honor of the Association. It was in debt, and its first duty was to take measures to relieve its executive officers from their embarrassments. He moved that every member be assessed a tax of two dollars to raise the necessary funds.

On motion, he was requested to prepare a subscription paper, and lay it upon the table for voluntary contributions.

*Female Physicians.*—Dr. Atlee, of Philadelphia, offered the following:

"*Whereas*, The subject of female education is exciting attention, and regularly educated female physicians have established themselves as practitioners of medicine; and

"*Whereas*, Female Medical Colleges, embracing all branches taught in other colleges, and all the conditions for graduation exist in the United States for the separate education of females; and

"*Whereas*, it is important that the standard of education and the observance of the code of medical ethics should be fostered and maintained by this Association, therefore

"*Resolved*, That the American Medical Association recognizes well-educated female physicians by the same laws that govern its own members."

Dr. Bowditch arose to a point of order, and reminded the President that the Association had postponed the order of the day for five minutes to allow Dr. Storer's question of privilege. He claimed that the five minutes had expired, and he moved to lay the resolutions on the table, which motion prevailed without a negative vote.

*Report on Insanity.*—Dr. C. A. Walker, of Boston, read the report of Dr. I. Ray, of Providence, R. I., on Insanity. It was an able and interesting paper.

Dr. Chipley, of Lexington, Ky., offered some remarks in vindication of the Superintendents of Insane Asylums, with reference to their connection with this Association. He referred to the fact that there were five such Superintendents present, who were a larger proportion of their class than the representatives of any other class of the medical profession present.

The report of Dr. Ray was referred to the Committee of Publication.

*Nominations and Place of Meeting.*—The Committee on Nomination of Officers and Place of Meeting, reported as follows:

*Place of Meeting*—New Orleans.

*President*—Samuel D. Gross, of Pennsylvania.

*Vice Presidents*—A. C. Post, of New York; John H. Atlee, of Pennsylvania; D. W. Yandell, of Kentucky, and H. R. Storer, of Massachusetts.

*Permanent Secretary*—William B. Atkinson, of Pennsylvania.

*Assistant Secretary*—J. G. Richardson, of New Orleans.

*Treasurer*—Casper Wistar, of Pennsylvania.

On motion of Dr. Davis, of Illinois, that portion of the report naming the place of meeting and officers resident there was laid on the table. The rest of the report was then adopted.

Dr. Davis remarked that the crisis had arrived, which he had long anticipated, when the matter of eating and drinking, and entertaining the Association had come to involve such an expense that no invitation had been extended in advance, by resident members of the profession, for the Association to meet in any city, and the Committee had reported New Orleans without an invitation from any one there. While there was no city in which he would like better to meet on personal accounts, and as a manifestation of re-union with the South, he felt that it would not be right to impose a meeting of the Association on that city. It was embarrassed in every relation of life, like all other places in that direction. It was impoverished, and though they would no doubt receive us with cordial and open hands, it would be wrong to tax them in that way. They could not view with the liberality and extravagance of Cincinnati; or, if they did, it would be at a sacrifice we should not admit of. He would give them another year to recover, and an opportunity to invite us. He therefore offered the following:

"*Resolved*, That the next annual meeting of the American Medical Association shall be held in the city of Washington on the first Tuesday in May, 1868, and every second year thereafter, until otherwise ordered by the Association.

"*Resolved*, That whenever the Association shall meet in the city of Washington, as directed in the above resolution, the Committee of Arrangements be strictly forbidden either to provide themselves, or accept provision by others, of any entertainment or excursion whatever."

Dr. Yandell, of Kentucky, arose, and was urged to take the stand, where he said:

"I have listened with pleasure, and derived some new light from the remarks of the gentleman from Chicago. I arise to explain the motives of the Committee in naming New Orleans, and in doing which I think I shall violate no confidence. When the place of meeting was called for, Dr. Horatio R. Storer, of Massachusetts, proposed New Orleans. The motion was seconded by Dr. Alonzo Palmer, of Michigan. As one of the few representatives from the South, I chanced to be in the Committee, and was asked my opinion in reference to New Orleans. I confess to you, gentlemen, that I never listened to a proposition in all my life which excited such emotions in my breast as this motion, coming from Massachusetts, and seconded by Michigan, to take this great body of brothers across the crimsoned waste of war and hold out the hand of fellowship to their brethren in the South. [Cheers.] I confess to you, that when Dr. Storer, of New England, and Dr. Palmer, of the mighty West, moved that we thus extend the hand of brotherhood to the medical profession in the South, I could not restrain my emotions from bringing tears of joy to my eyes.

"The first question was, whether it would be acceptable to New Orleans. I felt that I had some right to speak for the profession in New Orleans. My excellent father and myself had taught many of them as students of medicine. I had served with them, and was personally acquainted with most of those who had been in the Southern army. I knew they felt as I feel, that when peace came in 1865, we had been united again; in fact, that we had never been divided; that though politicians separated us by geographical lines, still the great republic of letters was one; that

"No pent-up Utica contracts our powers,
But the whole boundless continent is ours!"

"In the great republic of science all geographical lines should be obliterated. We of the South, and you of the North, the East, and the West, have a common heritage. The ashes of the illustrious Drake lie in your beautiful cemetery here. Have we of the South no claim to or interest in his name and his fame? Have you of the North no interest in the long list of great and good men who adorn the profession in the South? No interest in those whose labors have added glory not only to medicine but to the nation?

"Hence I said I believed every man in the South, in the land of flowers, of the mocking bird, and of beautiful women, waited but to have your hands extended to them to give you a welcome. [Cheers.] I said to the Committee that New Orleans was poor, that the whole South was poor, but that you would not be less welcome to sit under our vines and to take the fruit from our fig trees; that we could not give you such splendid entertainments as Cincinnati has done; but we could do what was better than eating and better than drinking; we could give you the warm grasp of the hand that you would take with you in memory to your homes. In view of this the Committee honored itself and honored New Orleans by selecting that place for its next meeting.

"I see the objection of Dr. Davis, but if he proposes to do no eating and no drinking at the next meeting, New Orleans is a better place than Washington city. [Applause.] If you want to put the Medical Association on low diet, go down there. [Cheers.] But the very reason of all others which should take the Association south of Mason and Dixon's line—if such a line there be now—is that the Southern people are not able to come to you. They are not here to-day because they could not afford to come. But they would welcome you to their homes. If you do not go to New Orleans, then go to some place within their reach."

Dr. Griswold, of Ohio, moved to substitute Knoxville, Tenn., instead of Washington city.

Dr. Sayre moved to substitute New Orleans, as in the original report. He thought that, with Dr. Davis's resolution of restriction as to entertainments, there would be no objection to that place.

Dr. Cox, of Maryland, indorsed all that had been said by his friend from Kentucky; he was proud that one man from the South had expressed himself as standing on the platform of the union of the medical profession. He never supposed there would be any difficulty in re-uniting the profession of medicine after the war was over. As to the entertainments of the Association, he was in favor of them, and thought if Dr. Davis's measure was adopted, the usefulness and attendance of the Association would be impaired. In England they did far more eating and drinking than was done here, and yet they accomplished a large amount of work.

Dr. D. H. Storer, of Boston, said the object of these meetings was to bring as many of the profession together as possible, and they should be held in such places as would accomplish that object. We should not meet where we have not been invited by resident members. The probability was we would be well received, but we ought to wait for a proper invitation. Ought we to go when we know there are a great many there who would wish us anywhere else?

---

FOURTH DAY.

Friday, May 10th, 1867. Dr. H. F. Askew, President, in the chair.

*Additional Delegates to the International Congress.*—The following were, on motion, elected additional delegates to the International Medical Congress to be held at Paris next August:—Drs. Wilson Jewell, of Pennsylvania; Ninian Pinkney, U. S. N.; John Hart, of New York; and Charles A. Pope, of Missouri.

Dr. Hibberd, of Illinois, then presented the following, which was carried:

*Whereas*, It has been officially announced that for the last two years the annual volume of the Transactions of this Association could be published only by the members of the Publication Committee becoming individually responsible for the cost of the same above the amount of funds in the treasury; and

*Whereas*, Such a condition of affairs is impolitic for the Association and unjust for the Committee; therefore

*Resolved*, That the Association does not expect the Committee on Publication to issue the volume of Transactions for the present year unless it can be done with the funds and the credit of the Association.

Dr. Hildreth submitted a resolution:

"That a Committee on Ophthalmology be appointed to report at the next session." Adopted.

Drs. Joseph S. Hildreth, of Chicago, Illinois, Henry D. Noyes and Cornelius R. Agnew, of New York, were appointed said Committee.

*The next Place of Meeting to be Washington, D. C.*—Dr. Davis's resolutions regarding the next place of meeting, etc., were then ordered from the table.

Dr. Hammar suggested St. Louis, Mo., as having favorable claims for the consideration of the Association.

After a lively debate, during which several amendments to the original resolution were entertained, the motion finally prevailed in the following form:

*Resolved*, That the next Annual Meeting of the American Medical Association shall be held in the city of Washington on the first Tuesday in May, 1868, and every second year thereafter, until otherwise ordered by the Association.

*Resolved*, That whenever the Association shall meet in the city of Washington, or elsewhere, as directed in the above resolution, the Committee of Arrangements be strictly forbidden either to provide themselves, or accept provision by others, of any entertainment or excursion whatever, which will conflict with the regular business of the body or its Sections.

*Cultivation of the Cinchona Tree.*—Dr. Atkinson read a communication from Dr. Henry F. Lyster, Secretary of the Wayne Co. (Mich.) Medical Society, requesting that some action be taken by the American Medical Association regarding the "introduction of the cinchona tree into the United States." For the sake of giving form to the discussion, he presented the subjoined:

*Resolved*, That a committee of three be appointed by the Chair, whose duty it shall be to memorialize Congress relative to the cultivation of the cinchona tree. Carried.

Drs. J. M. Toner and F. Howard, of Washington, D. C., and C. A. Lee, of Poughkeepsie, N. Y., were appointed said committee.

Dr. Atkinson read by title a paper from Dr. E. Harris, of N. Y., upon the "Causes of Cholera." Also another by Dr. E. Krackowizer, on "Local Anæsthesia."

Dr. Davis, in view of the fact that the hour of adjournment was rapidly approaching, offered a resolution which he thought would meet all objections.

*Resolved*, That such papers and reports as the several Sections have not been able to act upon, be referred to a special committee of *three*, to examine and act upon in all respects as is required in the proper Sections. Carried.

The Committee, as appointed, consisted of Drs. N. S. Davis, D. H. Storer and C. A. Lee.

Dr. Harris's and Dr. Krackowizer's papers were then, on motion, referred to said committee.

Dr. Atkinson read by title "Synopsis of an Essay on the Contagion, Infection, Portability and Communicability of the Asiatic Cholera in its relations to Quarantine. With a brief history of its origin and course in Canada, from 1832. By William Marsden, M. D."

Dr. Marsden made a few remarks in explanation of the objects, etc., of the paper.

Dr. Sayre moved to refer the paper to the Committee on Publication. Carried.

*Alterations in the Plan of Organization.*—The following was offered by Dr. Cox, of Maryland:

*Resolved,* That a committee of five be appointed by the Chair to take into consideration such amendments or alterations in the plan of organization of this Association, and to remedy defects, if any, and increase its efficiency, and report at the meeting in 1868. Adopted.

Drs. C. C. Cox, J. M. Toner, W. B. Atkinson, J. J. Woodward and John Shrady, were appointed in accordance with the above.

Dr. Davis moved that the resolution referring Dr. Marsden's paper to the Committee of Publication be re-considered. Carried.

The motion to refer said paper to the special committee, as previously provided, was then carried.

Dr. B. Howard, of New York, owing to the absence of the Secretary, read the report of the Surgical Section, which, after some corrections, was accepted.

*Cholera and Quarantine.*—Dr. Charles A. Lee, of New York, then submitted the following resolutions, bearing upon the subject of cholera, which were adopted as the sense of the Convention:

*Whereas,* it was declared by a vote of Congress, at its last session, that it is not within the constitutional powers of the General Government to establish a general and uniform system of quarantine for the different ports of the United States, and whereas, the cholera infection has been introduced into the United States, and did doubtless manifest itself in many of the cities, towns and villages of our country during the past season, and

*Whereas,* the experience of the city of New York, and other places, both at home and abroad, has demonstrated the efficacy of certain chemical disinfectants, especially *carbolic acid* and the *sulphate of iron,* in destroying or preventing the spread of cholera *virus,* it is hereby urgently recommended by this Association, that the attention of physicians of the United States be chiefly and constantly directed to the prompt and free use of such disinfectants wherever the cholera poison may show itself.

*Resolved,* That as the experience of Europe and the United States has satisfactorily shown that the cholera poison cannot be controlled or kept in check except where the *cordons sanitaires* are absolutely prohibitory of all intercourse, as was the case in the entire Island of Sicily, and the entire coasts and frontier of Greece, during the recent cholera epidemic, and,

*Whereas,* there is no good reason to believe that the people of the United States would not submit to the enforcement of such prohibitory measures, and non-intercourse, as is necessary to hold the cholera poison in check, especially after its introduction into the country, it is hereby recommended to all municipal bodies and boards of health to pay special attention to requisite sanitary measures, such as the cleansing of streets, lanes and alleys; the supply of pure drinking water to the inhabitants; the ample provision of chemical disinfectants, and their prompt employment in necessary cases; the separation of the sick from the healthy, in the same dwelling; the inspection and regulation of tenement houses; the provision of nurses, hospitals, and competent physicians for the sick poor, who may be attacked; provision for the early burial of the dead; the separation of the corpses from the living; and the prohibition of the custom of waking the dead, and all other measures which have been found necessary to control the progress of the disease.

*Resolved*, That experience proves that the publication of the facts connected with the existence and progress of cholera in any place, instead of disturbing the popular mind, while it reveals the exact extent of the danger, robs it of the hold of alarm and fear, with which the imagination surrounds indefinite pestilence, walking abroad by noon-day.

Dr. H. R. Storer read the minutes of the Section on Psychology, which were, on motion, referred to the Committee on Publication.

The reports of the Section on Practical Medicine, and on Meteorology, were read and disposed of in like manner.

The Committee on Nominations submitted their report as amended. The changes are:—*Committee of Arrangements*—Drs. Grafton Tyler, (Chairman,) William P. Johnson, F. Howard, Wm. Mawbury, Lewis Mackall, T. F. Many, J. M. Toner. *Assistant Secretary*—J. W. H. Lovejoy. Added to *Committee on Necrology*—Dr. Samuel Willey, of Minnesota, and Dr. Samuel M. Welch, of Galveston, Texas.

*Remuneration of Permanent Secretary.*—Dr. M. A. Pallen presented the subjoined:

*Whereas*, it was the intention of the resolution originally introduced, creating the office of Permanent Secretary, to pay said officer a certain sum annually, as a salary for services as such; and whereas, Dr. William B. Atkinson, our present efficient and urbane Secretary, has never received any money whatsoever in payment of said services, therefore be it

*Resolved*, That the Permanent Secretary hereafter, and from this date, be authorized to draw a warrant upon the Treasurer for the expenses incurred in his attending each meeting of the Association, and that the Treasurer is hereby instructed to pay the same. Unanimously adopted.

Dr. Atlee moved that the thanks of the Association be tendered to the Permanent Secretary for past services. Carried.

*The Annual Assessment.*—Dr. Toner proposed the following, which includes an article of the Constitution, with the amendments added; The sum of five dollars shall be assessed annually upon each delegate to the sessions of the Association, as well as upon each of its permanent members, whether attending or not, for the purpose of raising a fund to defray the *necessary* expenses of the Association, and for printing the Transactions. The payment of this assessment shall be required of the delegates and members in attendance upon the sessions of the Association previously to their taking seats and participating in the business of the session. Permanent members not attending shall forward their yearly dues to the Treasurer, and thereby shall be entitled to receive a copy of the printed Transactions, the same as delegates. Referred, after an animated debate, to Committee on Revision of the Constitution and By-Laws.

Dr. Hibberd asked that Dr. H. R. Storer be permitted to use, in the preparation of a paper, certain matter previously presented by himself to the Association. Permission granted.

Votes of thanks to various Railroad Companies, and others, for courtesies extended the Association, were then passed.

Dr. Hibberd's resolutions, regarding the use of unofficinal preparations, and the relations between the profession and the venders of nostrums, were then called up.

Dr. Post called attention to the proper distinctions between the terms "unofficinal" and "magisterial."

Dr. Cox, as an amendment, desired to insert after *manufacturing*, the words "advertising or selling quack medicines or nostrums." Lost.

Dr. Bibbins moved the reference of the whole subject to the Committee on Revision of the Constitution, etc. Carried.

*Female Education again.*—Dr. Atlee then pressed his resolutions on the subject of female medical education. A motion to take them from the table was carried by a vote of 56 to 52.

Dr. Pallen, of Missouri, was opposed to the discussion of the subject. Women were not by nature fitted for the practice of medicine. It had been tried in Europe, and had proved an utter failure. Ladies possessed of any delicacy could not acquire the proper amount of knowledge. Imagine a young lady, with gigantic chignon and garbed in silks, entering the charnel-house, and bending over a corpse, microscope in hand, searching for cancer cells, etc., etc.

Dr. Davis thought the discussion of the subject, at this time, would only furnish newspaper gossips with a topic, and could do no possible good. He therefore moved to refer the whole subject to the Committee on Medical Ethics.

Dr. Bowditch, of Mass., was opposed to this way of disposing of such an important matter. He had moved yesterday to lay the resolutions on the table, simply because he thought the Convention was not then prepared to act upon them. The question had nothing to do with the laws of nature or the manner in which ladies were to acquire the proper amount of knowledge. The question was simply whether or not they should be recognized when they *had* acquired that knowledge, as many of them undoubtedly had. The Doctor mentioned several instances in which the practice of medicine by lady physicians had been attended with great success.

Dr. Davis's motion to refer to Committee on Medical Ethics was carried by a large majority.

Dr. Hibberd moved that Dr. Theophilus Parvin, of Indiana, be appointed to render a special report on the Surgical Diseases of Women, at the next annual meeting. Adopted.

A vote of thanks was tendered to Mr. F. Hopkins for the free use of his hall.

A communication from Dr. J. Homberger, expressing his desire to resign from the Association, was received, and finally referred to Committee on Medical Ethics.

*The Provision for Chronic Insane.*—After a resolution, offered by Dr. S. C. Hughes, thanking the Press for impartial reports of the proceedings, Dr. C. A. Lee read the following:

*Resolved*, That providing for the poor chronic insane in the jails and almshouses of our country, as at present practiced in nearly all the States of the Union, is a gross violation of the laws of humanity, and contrary to the Divine injunction of "doing to others as we would be done by."

*Resolved*, That where the regular hospitals for the insane of a State are insufficient to accommodate both acute and chronic cases that are sent to them, this Association would strongly recommend the procurement of a suitable amount of land in the vicinity, and the erection of convenient, well-planned, and well-ventilated, but comparatively inexpensive buildings, in connection with and under the same general supervision as the hospitals themselves, where those who are able to labor, and would be benefited by light and regulated employment, may be suitably accommodated and properly cared for.

*Resolved,* That the example of Massachusetts in establishing asylums for the accommodation and humane treatment of the chronic insane, is worthy of all praise and imitation, and in the opinion of this Association, such institutions, if rightly inaugurated and judiciously carried on, will be a benefit to the State in an economical point of view, will raise the character of the State Hospitals, and will greatly subserve the interests of the insane generally.

*Resolved,* That as the present insane hospitals are capable of accommodating but a small proportion of the 40,000 insane of the United States, and as almshouse and jail provision is not adapted to their proper care and treatment, this Association would recommend to the proper State authorities to make such further provision in the direction above indicated as may tend to the amelioration of their condition, if not the restoration of their rational and moral faculties. Adopted.

Dr. Bibbins moved to refer to a special committee of five, to report at the next annual meeting. Carried.

Drs. C. A. Lee, New York; Guntry, Ohio; John Fonerdin; Walker, Mass.; Chipley, Ky., were appointed said committee.

*The late Surgeon C. S. Tripler, U. S. A.*—Dr. Cox submitted the following resolutions, which were unanimously adopted:

*Resolved,* That in the loss of Surgeon Charles S. Tripler, U.S.A., who died in this city since the last meeting of the Association, the profession throughout the country, the Army of the United States, and the Society especially, have experienced a serious loss.

*Resolved,* That in the high moral integrity, Christian character, professional ability, and conscientious love of his vocation, we recognize in Dr. Tripler one of the truest illustrations of a sound physician and a good man.

*Resolved,* That the condolence and sympathies of this Association are hereby tendered to the family and relations of the deceased; and the Secretary is directed to communicate to them a copy of these resolutions.

Dr. Davis moved that the committee charged with procuring suitable accommodations for the Association meetings in the Smithsonian Institute, in Washington, D. C., be continued. Carried.

Dr. Alden March, of New York, offered the following:

*Resolved,* That the thanks of the Association are due, and are hereby tendered to the President and retiring officers for the ability, impartiality and courtesy manifested in the discharge of their arduous duties. Carried.

Dr. Cox moved that the surplus copies of the Transactions of the Association, not yet out of print, be sent to the Secretaries of similar organizations in exchange for the volumes published by their own bodies. Carried.

Dr. Hughes presented the following:

*Resolved*, That those members of the Association who have contributed to the amount of five dollars to the publishing of future Transactions, shall be entitled to any back volumes of the Transactions to the amount of the same, as they may want. Carried.

After the passage of several votes of thanks, the meeting adjourned at two P. M., to meet at the time and place previously designated.—[*Extracted from Boston Medical Journal.*

